# High Dietary Intake of Rye Affects Porcine Gut Microbiota in a *Salmonella* Typhimurium Infection Study

**DOI:** 10.3390/plants11172232

**Published:** 2022-08-28

**Authors:** Julia Hankel, Bussarakam Chuppava, Volker Wilke, Clara Berenike Hartung, Uthayakumar Muthukumarasamy, Till Strowig, Knud Erik Bach Knudsen, Josef Kamphues, Christian Visscher

**Affiliations:** 1 Institute for Animal Nutrition, University of Veterinary Medicine Hannover, Foundation, 30559 Hannover, Germany; 2 Helmholtz Center for Infection Research, 38124 Braunschweig, Germany; 3 Hannover Medical School, 30625 Hannover, Germany; 4 Department of Animal Science, Aarhus University, 8830 Tjele, Denmark

**Keywords:** dietary fiber, arabinoxylan, fructan, foodborne pathogen, zoonosis, *Bifidobacterium*, *Faecalibacterium*

## Abstract

Bacterial fermentation of undigested carbohydrates in the hindgut has considerable potential for the stimulation or inhibition of the growth of distinct bacteria within microbiota. The aim of the present study was to evaluate whether high levels of rye affect porcine gut microbiota composition with subsequent effects on the load of *Salmonella* Typhimurium, an intestinal pathogen with zoonotic relevance. Therefore, forty-two 25-day-old piglets were allocated to two groups and fed a diet containing either 69% wheat or 69% rye for 35 days. One week after introducing the two different diets, the piglets were experimentally infected with *Salmonella* Typhimurium. The microbiota composition of cecal and fecal samples of the piglets were evaluated 28 days after infection. In the cecum, promoted growth of *Bifidobacterium*, several lactic acid bacteria and *Faecalibacterium prausnitzii* were seen in pigs fed the diet containing 69% rye. Bacterial species belonging to the genera *Bifidobacterium* and *Catenisphaera* were associated with differing bacterial counts of *Salmonella* Typhimurium detected in the cecal contents of all piglets in both feeding groups via cultural cultivation. The high intake of rye instead of wheat seems to promote the growth of beneficial intestinal bacteria accompanied by impaired growth conditions for the foodborne pathogen *Salmonella* Typhimurium.

## 1. Introduction

Carbohydrates represent a diverse group of simpler monosaccharides and disaccharides to complex organized polysaccharides which form cell walls [1]. Depending on their chemical form (e.g., linkages between carbohydrate components), carbohydrates are degraded by either endogenous or microbial enzymes, thus having a great impact on the site of their digestion and how they influence the physiology of the gastrointestinal tract [2]. However, a classification based purely on chemistry does not allow a simple translation into nutritional properties, and terminology based on physiological properties would help to focus on the aforementioned potential nutritional properties [2]. As neither chemical nor physical description of carbohydrates directly reflects their physiological properties and health benefits, a number of terms to describe physiological functions of carbohydrates have been created, one of which is dietary fiber [3]. Dietary fiber is defined as carbohydrate polymers with a degree of polymerization not lower than three, which are neither digested nor absorbed in the small intestine [3]. This component has, compared to other dietary components, the main modulating effects on gastrointestinal microbiota and environment and on the production of fermentation products [2]. The bacterial fermentation of undigested carbohydrates depends on its physiochemical properties as well as the fiber dosage and the bacterial community composition of the individual consuming the fiber [4]. 

Cereal grains from different species differ significantly in several carbohydrate fractions, while rye has the highest concentrations of soluble arabinoxylans and fructans [5]. Cereal grains such as wheat and barley are frequently used as main ingredients in pig diets [6,7]. Comparing rye with wheat, the carbohydrate composition of rye is similar to wheat in many aspects, but rye has a higher content of fructans, arabinoxylans and mixed linked β(1→3,1→4)-D-glucan (β-glucan) than wheat. Arabinoxylans are the major part of dietary fiber polysaccharides and of soluble dietary fiber in rye [8]. The arabinoxylan molecules are broken down by enzymes that are not present in the endogenous digestive juice secreted in the gastrointestinal tract or present on the brush borders of the small intestine epithelium in monogastric species; however, intrinsic enzymes are present in the grain or can be produced by bacteria within the gastrointestinal tract [8]. This complex molecular structure of rye arabinoxylans makes them resistant against the degradation of the residing microbiota of the stomach and small intestine of monogastric species [8]. Arabinoxylans from rye as well as from wheat are almost completely recovered in ileal effluents and only modestly reduced in size during passage through the small intestine, therefore having a much higher influence on the viscosity in the small intestinal digesta [8]. The ileal digestibility of arabinoxylans fed a rye-based diet compared to a wheat-based diet to pigs is higher (19.2% vs. 8.2%) [9]. In addition, the ileal digestibility of starch and amino acids in growing pigs is greater when offered wheat compared to hybrid rye [10]. Dietary components that are not digested by endogenous enzymes in the small intestine become available as substrates for the microbiota present in the large intestine [2]. Finally, whole grains are a rich source of phytochemicals, and the most important classes of phytochemicals found in cereals of common wheat or rye differ [11,12]. Concentrated in the bran fraction, wheat has a high phenolic content, principally alkylresorcinols and hydroxycinnamic acids (ferulic, sinapic and ρ-coumaric acids) [12]. However, rye contains still more alkylresorcinols compared to wheat and the other cereal varieties, which is related to the high level of folate in this grain [12]. Other phytochemicals such as tocols, polyphenols and ferulic acid are found at lower levels in rye compared to wheat [12]. Phytochemicals undergo microbiotic metabolism before absorption and might also affect the composition of the microbiota [11,12].

As the consumption of dietary fiber promotes extensive metabolic interactions among intestinal bacterial species, there is considerable potential for the indirect stimulation of the growth of other microbes within the community through the utilization of by-products of other community members, a process called cross-feeding [4]. It seems that only a limited number of versatile polysaccharide-degrading bacterial species are present in the large intestine and degrade arabinoxylans to smaller molecules, which are cross-fed to lactobacilli and bifidobacteria [8]. In addition, bacterial fermentation of polysaccharides results in the production of acidic fermentation end products, primarily lactic acid and short-chain fatty acids (SCFA), which reduce intestinal chyme’s pH value [4]. SCFAs are being considered as potential prebiotic substances to control *Salmonella enterica*, but also have multiple effects on *Salmonella enterica*’s virulence genes [13]. Probiotics and prebiotics, for example, are proposed to reduce bacterial pathogens by increasing SCFA levels in the intestinal tract. Nevertheless, alterations in SCFA concentrations and ratios that favor acetate and decrease total amounts of propionate and butyrate might make the organisms more prone to invasion [14], as indicated by the invasion gene expression and virulence of *Salmonella* Typhimurium, which are altered by SCFA concentration and composition [14]. Thus, it is intended to favor the proportion of butyric acid among the SCFA. While pathogenic properties of *Salmonella* spp. have been studied quite extensively, it is only recently that the interactions of *Salmonella* spp. with gut microbiota, colonization resistance and ways to overcome this resistance have been the focus of investigation [15].

*Salmonella enterica* subspecies *enterica* serovar Typhimurium (*Salmonella* Typhimurium) can be carried by pigs asymptomatically in their tonsils, gut and gut-associated lymphoid tissue for months [16]. *Salmonella* Typhimurium is a foodborne pathogen. Zoonosis monitoring activities carried out in 2019 in 36 European countries revealed 87,923 confirmed human salmonellosis cases, this being the second most commonly reported gastrointestinal infection in humans, and an important cause of foodborne outbreaks [17]. Pig meat and products thereof are one of the four most implicated food vehicles [17].

As the bacterial fermentation of undigested carbohydrates in the hindgut has considerable potential for influencing intestinal microbiota composition, we hypothesized that high dietary levels of rye affected porcine gut microbiota with subsequent effects on the intestinal load of *Salmonella* Typhimurium, which may clarify the underlying cause of the observed lower *Salmonella* counts in the gut when pigs were fed a diet with a high proportion of rye [18]. Therefore, the aim of the present study was to evaluate the intestinal microbiota composition of piglets under the implemented feeding measures and to highlight possible interactions between *Salmonella* Typhimurium and the gut microbiota. The overall objective of the investigation was to find new ways to reduce the *Salmonella* load in the intestines of pigs prior to slaughter, which should reduce the risk of contamination, thus being relevant in a food safety context.

## 2. Materials and Methods

The animal study protocol was approved by the Ethics Committee on Animal Experiments of the Lower Saxony State Office for Consumer Protection and Food Safety, LAVES (Niedersächsisches Landesamt für Verbraucherschutz und Lebensmittelsicherheit; reference: 33.8-42502-04-19/3257, 27.11.2019).

### 2.1. Experimental Design

In three independent repetitions (Exp1, Exp2, Exp3), a total of forty-two weaned piglets (aged 24.9 ± 0.7 days at the start of the experiments) of one farm (Farm for Education and Research in Ruthe, University of Veterinary Medicine Hannover, Foundation) were allocated to two groups and housed individually in a biosafety level 2 animal facility (pen dimensions per animal: 1.1 m × 2.2 m). With the aim of creating comparable groups, the piglets were selected on the basis of their body weight (control groups: 7.48 ± 1.11 kg and experimental groups: 7.48 ± 1.20 kg), sex ratio (female/male castrated) and sow (siblings distributed in both groups). As a parental lines served db.Viktoria and db.77^®^ of BHZP genetics (BHZP GmbH, Dahlenburg-Ellringen, Germany). The piglets were housed under a 12 h light, 12 h dark lighting schedule. The room temperature was recorded daily and amounted on average to 24.5 ± 0.8 °C (in a range of 28 °C initially and 21 °C at the end of the study). The piglets had ad libitum access to feed and water. They were fed a complete pelleted feed based on either 69% wheat (control diet) or 69% hybrid rye (variety Trebiano, KWS LOCHOW GmbH, Bergen, Germany; experimental diet).

After a seven-day adaptation period, all piglets were orally infected with 2 mL of a broth containing ~ 1 x 10^7^ colony-forming units (CFU) of *Salmonella* Typhimurium. The used *Salmonella* serotype was obtained from a field study by Visscher et al. [19]: *Salmonella* Typhimurium 1,4,5,12:i:1,2; DT 193. The bacterium was grown aerobically onto Columbia blood agar (Oxoid^TM^ Deutschland GmbH, Wesel, Germany) at 37 °C for 16 to 24 h. An isotonic 0.9% sodium chloride solution was used as the basis for the infection bouillon. A defined density of *Salmonella* Typhimurium was adjusted (0.5 McFarland units) using a densitometer (DEN 1B, biosan SIA, Riga, Latvia), which already corresponded to the targeted challenge dose. The challenge dose was verified by plating dilutions of bacterial culture and quantifying colonies. After 28 days postinfection, piglets were dissected. Prior to the dissection, the piglets were anaesthetized with neuroleptanalgesia by means of a combination of ketamine (15 mg/kg body weight intramuscularly) and azaperone (2 mg/kg body weight intramuscularly). Subsequently the pigs were euthanized by intracardial injection of T 61 (0.6 mL/10 kg body weight). The abdominal cavity was opened along the linea alba. The caecum was preplaced, double-ligated and then removed. Cecal chyme and fecal samples (obtained per rectum) of the pigs were collected without delay. Within the same sample *Salmonella* counts and microbiota composition were determined. Until the microbiota analyses, the samples were stored at −80 °C.

### 2.2. Diet

The remaining 31% of both diets, the control (69% wheat) and the experimental diet (69% rye), mainly consisted of soybean meal, barley, potato protein, minerals, soybean oil and feed additives. Piglets had free access to water.

The chemical composition of the diets were analyzed by standard procedures in accordance with the official methods of the Association of German Agricultural Inspection and Research Institutes (Verband Deutscher Landwirtschaftlicher Untersuchungs- und Forschungsanstalten; VDLUFA) [20]. Carbohydrate and lignin content were analyzed at the Department of Animal Science, Aarhus University, Denmark by standardized methods as described in Jaworski et al. [21]. A detailed list of ingredients and the chemical composition of the diets can be taken from Table 1. The diets were comparable in their energy, crude protein and crude fiber contents, but differed in their contents of certain carbohydrate fractions.

The carbohydrate composition of the compound feeds is shown in Table 2. The concentration of insoluble noncellulosic polysaccharides (I-NCP) was greater than the concentrations of soluble noncellulosic polysaccharides (S-NCP) in both diets with values (percentage of total nonstarch polysaccharide (NSP)) in the order of the control diet (52.8%) and 53.3% for the experimental diet.

The concentration of S-NCP was higher in the experimental diet, reaching a value of 4.6% (dry matter basis) compared to the control diet (3.5% on dry matter basis). The major sugar monomers in S-NCP were arabinose, xylose and glucose. Xylose and arabinose mainly originate from soluble arabinoxylans, whereas the majority of glucose originates from soluble β-glucans [21]. As a percentage of total NSP, the control diet (28.4%) still contained less S-NCP compared to the experimental diet (32.8%). Similar to the diets based on wheat and rye breads used in the investigations of Bach Knudsen et al. [9], the diets of the present study were comparable in dietary fiber (control diet: 14.3% and experimental diet: 15.6%) but with different proportions of the main dietary fiber polymers: in wheat, cellulose and in rye, arabinoxylans (arabinose + xylose + uronic acids). The wheat-based diet had a higher total content (2.4%) and a higher proportion (cellulose accounted for 19.4% of total NSP) of cellulose compared to the experimental diet (1.8% and 13.0%). The rye-based diet had a higher content of arabinoxylans (8.1%) and glucose (= soluble β-glucan [21], 2.6%) compared to the control diet (7.2% and 1.5%). While the proportions of arabinoxylans were similar (arabinoxylans represented 57.7% of total NSP in the control diet and 58.1% in the experimental diet), the proportions of glucose differed between the diets (glucose represented 12.2% of total NSP in the control diet and 18.4% in the experimental diet).

### 2.3. Microbiological Analysis

*Salmonella*’s quantitative analysis of the fecal samples and cecal contents was performed following the DIN EN ISO 6579-2 guidelines [22] as described in Chuppava et al. [18]. In brief, *Salmonella*’s quantitative analysis was performed using the most probable number (MPN) method. One gram of homogenized feces or cecal contents and 9 mL of buffered peptone water (BPW) were vortexed. The bacterial count of the sample material was determined by further serial dilution with BPW in triplicate. After a 24 h incubation at 37 °C, the total volume of each well was transferred to a deep well block filled with modified semisolid Rappaport–Vassiliadis (MSRV) agar and incubated for 24 h at 41.5 °C. The results were confirmed by cultural cultivation on Brilliance Salmonella agar. Subsequently, the number of bacteria was calculated with the help of an MPN software program [23]. 

### 2.4. DNA Extraction, Sequencing and Data Processing

First, DNA was extracted from samples using the ZymoBIOMICS 96 MagBead DNA Kit (Zymo Research Europe GmbH, Freiburg, Germany). The hypervariable region V4 of the 16S rRNA gene was amplified in accordance with previously described protocols [24]. The primer F515/R806 was used. Amplicons were sequenced on the Illumina MiSeq platform (PE300), while the Usearch8.1 software package was used to assemble, quality control and cluster the obtained reads. The command “fastq_mergepairs” with the argument “fastq_maxdiffs 30” was used to merge the reads. Chimeric sequences were identified and removed with the help of the command “cluster_otus” (-otu_radius_pct 3) and the Uchime command included in the Usearch8.1 workflow. Quality filtering was set up with “fastq_filter” (-fastq_maxee 1) according to a minimum read length of 200 bp. Reads were clustered into 97% ID operational taxonomic units (OTUs). The UPARSE algorithm [25] was used to determine the OTU clusters and representative sequences. Silva database v128 [26] and the RDP Classifier [27] were used for taxonomic assignment with a bootstrap confidence cut-off of 70%.

Samples with fewer than 999 total reads were removed. Chloroplast and mitochondria as well as OTUs that were not present in at least more than one sample and OTUs with an abundance <0.02% were pruned. After the filtering steps, all 84 samples were included in the statistical analyses. The dataset contained 1,517,733 reads (mean number of reads: 18,068; range: 9308–24,427) mapped to 319 operational taxonomic units (OTUs).

### 2.5. Statistical Analysis

R (version 4.0.2) and the R-package “phyloseq” (version 1.32.0, [28]) were used for data visualization and analyses. Selected alpha diversity indices (Observed, Chao 1, and Shannon) were also calculated with “phyloseq”. The means of alpha diversity estimates were compared, with the aim to evaluate the influence of the factor treatment (Table S1). Data were checked for normality by analyzing the model residuals with the Shapiro–Wilk normality test before pairwise comparisons were conducted, all implemented in the package “rstatix” (version 0.6.0, [29]). The statements of statistical significance were based upon *p*-values < 0.05. The total community structure and composition of samples were assessed for changes in relation to experimental repetition (Exp1, Exp2, Exp3) and diet by a permutational multivariate analysis of variance using the Bray–Curtis distance (PERMANOVA) via the adonis function of the “vegan” package (version 2.5.6, [30]). Ordination was performed using the Bray–Curtis dissimilarity-based principal coordinate analysis (PCoA). Differentially abundant OTUs between both treatment groups were identified with the help of the R package “DESeq2” (version 1.28.1), which uses tests based on the negative binomial distribution [31]. This evaluation was done over all consecutive identically performed repetitions and additionally separated for each trial. Raw *p*-values were adjusted using the method of Benjamini and Hochberg [32] to control a false discovery rate (FDR) of 5%. Additionally, a cut-off for the log2-fold change of ±1 was set. A Venn diagram was generated using the package “rstatix” to show significantly different OTUs between pigs fed the two different diets and those that were shared among sampling sites: cecal contents and feces. To evaluate whether the differences between the two feeding groups were related to *Salmonella* counts, all 42 piglets were divided into two new groups according to whether the *Salmonella* counts in cecal contents were above or below the average value: >3.2 log_10_ CFU/g sample (n = 20); <3.2 log_10_ CFU/g sample (n = 22). Afterwards, differentially abundant OTUs between both groups were identified with the help of the R package “DESeq2” as well and results were visualized with ggplot2 (version 3.3.5 [33]).

## 3. Results

Results related to animal performance have already been evaluated and published in Chuppava, et al. [18]. Feeding a diet containing 69% rye to young pigs was not associated with significant differences in performance, i.e., average body weight gain, feed intake and feed conversion ratio compared with pigs fed with generally high amounts of wheat. Neither experimental infection of pigs with *Salmonella* Typhimurium, nor the supply of rye instead of wheat resulted in visible abnormalities in fecal consistency.

### 3.1. Gut Microbiota Diversity and Microbial Community

**Figure 1:**
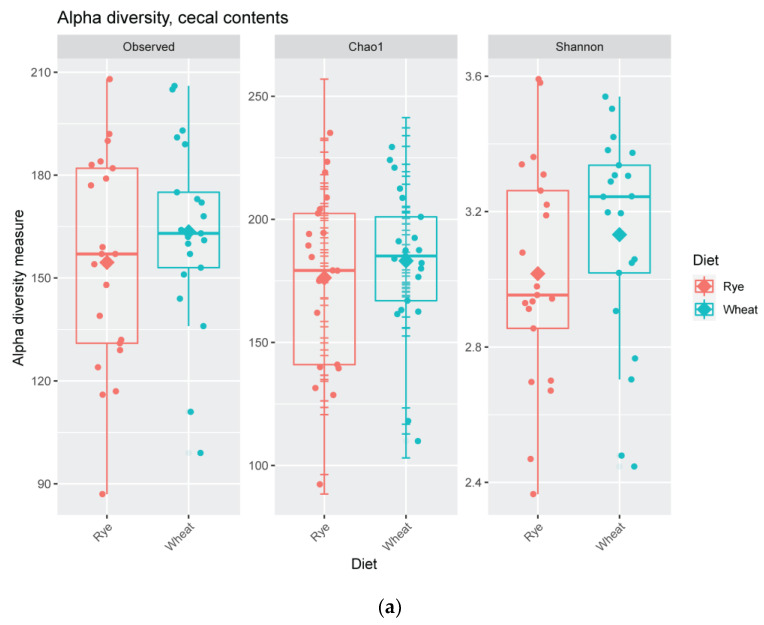

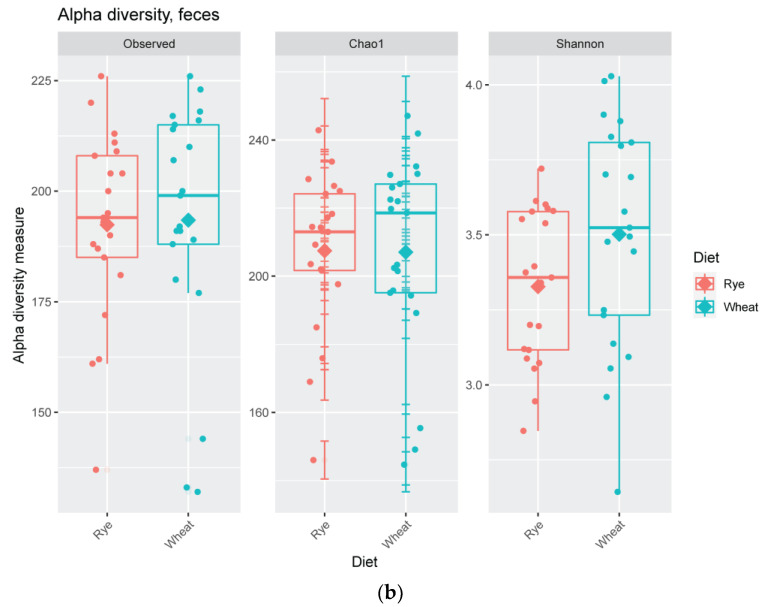
Boxplots showing Observed species, Chao1 and Shannon index in (**a**) cecal contents and (**b**) feces of pigs depending on diet.

The use of rye instead of wheat in the pigs’ diet had no influence on microbiota diversity, neither in cecal contents nor in fecal samples of pigs (Table S1, Figure 1).

**Figure 2:**
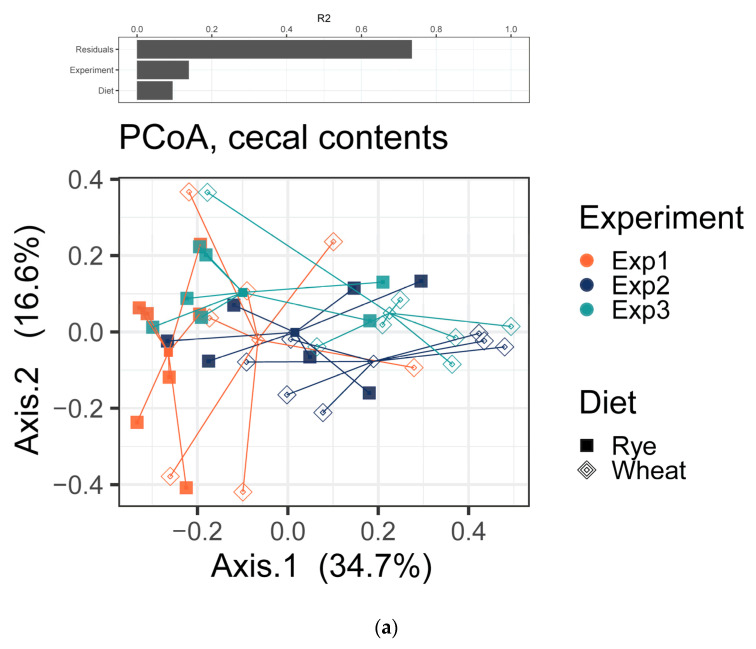

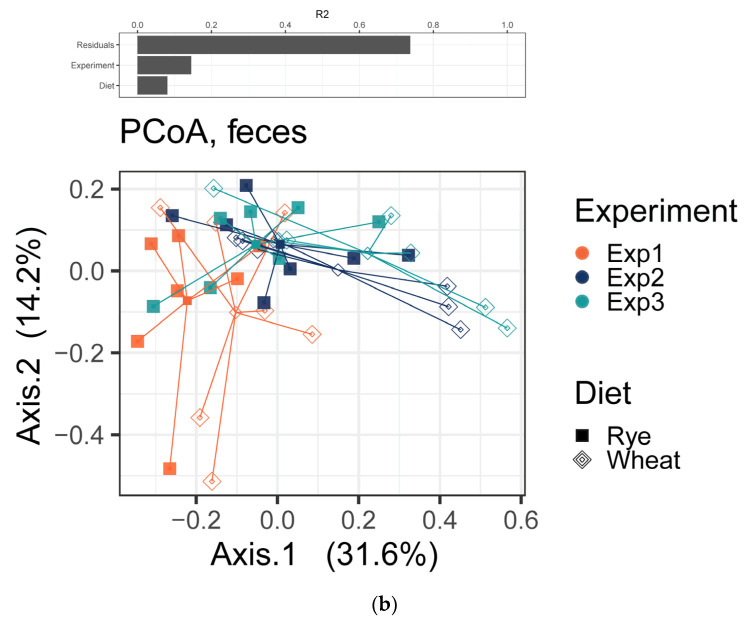
A permutational multivariate analysis of variance (PERMANOVA) on Bray–Curtis distances was used to quantify the contribution of the factors Experiment and Diet to the differences in microbial composition of the samples (above). A Bray–Curtis dissimilarity-based principal coordinate analysis (PCoA) was performed on (**a**) cecal contents and (**b**) feces of pigs fed with either rye- or wheat-containing diets. Different colors represent samples obtained from different experiments. Different point shapes represent samples of pigs receiving different diets. Lines connect samples obtained from the same groups (below).

The bacterial composition of the cecal contents and feces of pigs differed significantly (R^2^ = 0.07573, *p* = 0.001, Figure S1). The factor diet had a significant effect on the bacterial composition of the samples in cecal contents as well as in feces, while samples separated more clearly in cecal contents (R^2^ = 0.095) than in feces (R^2^ = 0.081) by this variable alone (Table S2, Figure 2).

### 3.2. Identification of Specific Taxa Associated with the Diet

**Figure 3:**
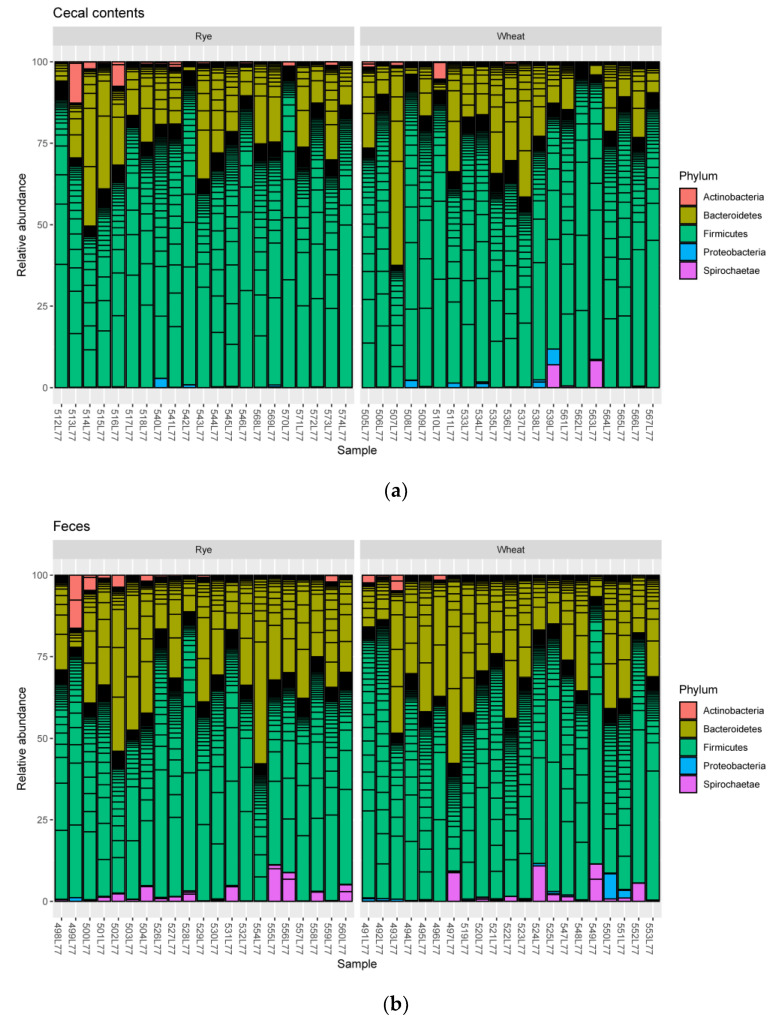
Bar charts represent the relative abundances of the dominant phyla in (**a**) cecal contents and (**b**) feces of pigs.

Microbiota of cecal contents were dominated at the phylum level by *Firmicutes* (76.6% ± 13.0) and *Bacteroidetes* (20.6% ± 12.8), followed by *Actinobacteria* (1.2% ± 2.60). Fecal microbiota was dominated by *Firmicutes* (65.1% ±10.4) and *Bacteroidetes* (30.0% ± 11.4) as well, followed by *Spirochaetae* (2.27% ± 3.10) and *Actinobacteria* (1.14% ± 2.71). The relative abundances of bacterial phyla in every sample are shown in Figure 3.

A total of 21 differentially abundant OTUs were identified in the pairwise comparisons of pigs fed the two different diets in cecal contents, 15 OTUs in feces. All those differentially abundant OTUs in the pairwise comparisons of the groups that fulfilled the set criterion (Benjamini and Hochberg adjusted *p*-values <0.05 and cut-off for the log2-fold change of ±1) can be found in Table S3 (cecal contents) and Table S4 (feces).

**Figure 4:**
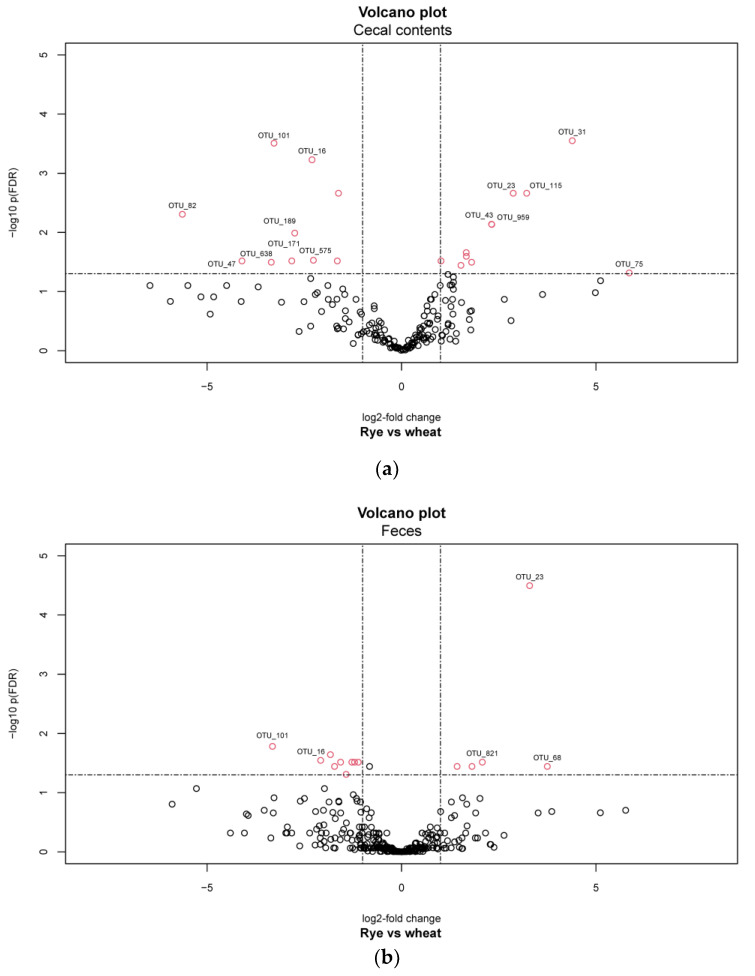
Volcano plot visualizing –log10 FDR-adjusted *P*-values versus log2-fold changes for all 319 OTUs. The horizontal lines show significance threshold for FDR < 0.05. Each open point represents a single OTU in: (**a**) cecal contents and (**b**) feces. Open points above the significance threshold and beyond the log2-fold change criterion of ±1 are indicated in red, while OTU names are labeled beyond the log2-fold change criterion of ±2.

Only in cecal contents, OTU_31 was enriched in pigs fed rye (Figure 4). This OTU represents a bacterial species that is assigned to the genus *Bifidobacterium*, within the phylum *Actinobacteria* (the relative abundance of OTU_31 in the cecal contents of pigs fed wheat was 0.56% ± 0.44 and 1.21% ± 2.59 in pigs fed rye). Besides this bifidobacterial strain, one strain belonging to the genus *Catenisphaera* of the family *Erysipelotrichaceae* (OTU_23) and four further bacterial strains belonging to the order *Lactobacillales* with genera comprising lactic acid bacteria [34] were enriched in the cecal samples of piglets fed the experimental diet: two of the genus *Lactobacillus* (OTU_115 and OTU_43) and two of the genus *Streptococcus* (OTU_959 and OTU_2). In addition, OTU_40 was significantly enriched in the samples of pigs fed the experimental diet (the relative abundance of OTU_40 in the cecal contents of pigs fed wheat was 0.65% ± 0.47 and 1.01% ± 0.73 in pigs fed rye). This OTU represents a bacterial species that is assigned to the genus *Faecalibacterium,* with one identified species *Faecalibacterium prausnitzii* that clusters in different genospecies [35].

**Figure 5:**
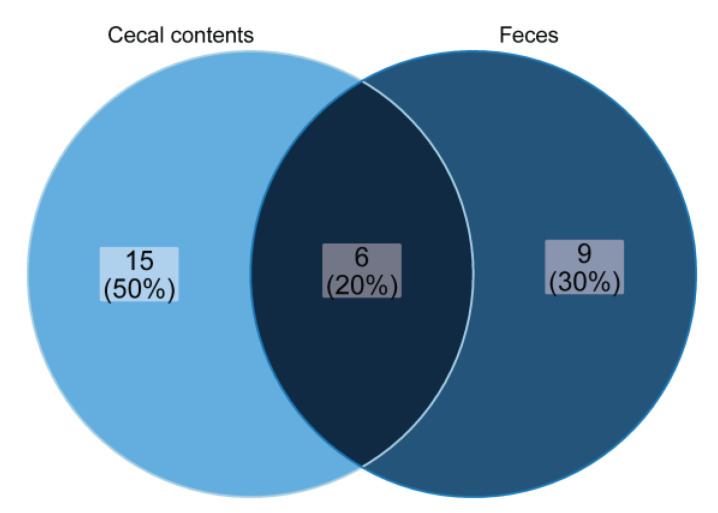
Venn diagram showing significantly different OTUs (selected with a criterion of FDR-adjusted *P*-values <0.05 and absolute log2-fold change >±1) between pigs fed different diets that are shared in cecal contents and feces.

A total of 36 OTUs differed between the feeding groups in cecal contents (21 OTUs) and in feces (15 OTUs). Six of those thirty-six OTUs already differed significantly in cecal contents between pigs fed different diets and continued to differ in further parts of the hindgut, as shown in fecal samples. Those six OTUs comprised OTU_101 (genus *Romboutsia* of the order *Clostridiales*), OTU_16 (genus *Terrisporobacter* of the order *Clostridiales*), OTU_6 (genus *Turicibacter* of the order *Erysipelotrichales*), and OTU_23 (genus *Catenisphaera* of the order *Erysipelotrichales*), OTU_2 (genus *Streptococcus* of the order *Lactobacillales*) and OTU_13 closely matching the family *Prevotellaceae*. Sequences of OTU_101, OTU_16 and OTU_6 were lower in the cecal contents and feces of pigs fed the experimental diet, while sequences of OTU_23, OTU_2 and OTU_13 were enriched. A total of 50% of the OTUs, which were previously different in the cecum, showed no more differences in the feces. However, nine additional OTUs in feces appeared to be different between pigs fed different diets (Figure 5).

Despite performing consecutive repetitions under the same conditions, the PERMANOVA test on Bray–Curtis dissimilarities revealed that the factor experimental replication contributed significantly to the differences in microbial composition in cecal contents as well as in feces (Table S2). When evaluating the trials separately, it became clear that OTU_31, which was found to be clearly enriched when comparing the feeding groups over all three trials together, differed significantly only in experiment two and experiment three. In contrast, OTU_23 differed significantly in all three independent repetitions (Tables S5–S7).

**Figure 6:**
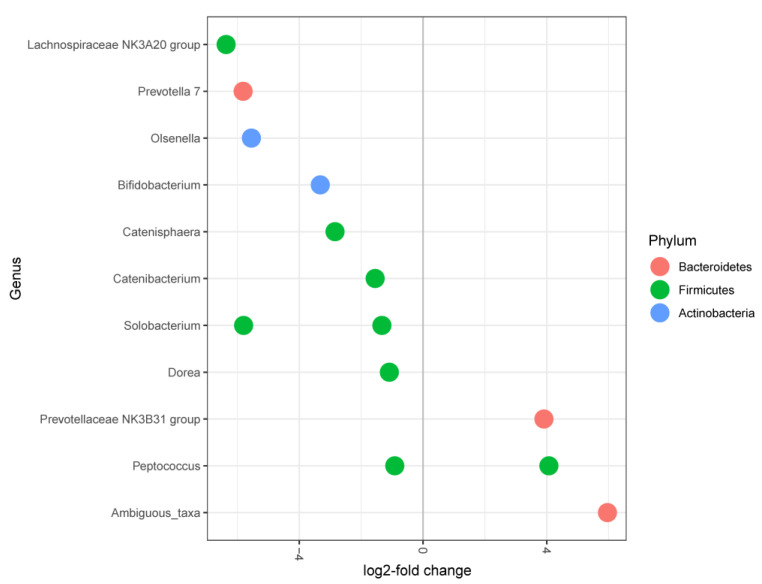
DESeq2 analysis of differentially abundant OTUs (significance threshold for alpha < 0.01) of piglets with *Salmonella* counts above versus below 3.2 log_10_ CFU/g cecal content. Each point represents a single OTU grouped by genus and by color according to which taxonomic phylum the OTU originates from.

*Salmonella* counts in cecal contents (control: 3.34^a^ ± 0.50 log10 CFU/g; experimental: 3.08^b^ ± 0.56 log10 CFU/g) and feces (control: 3.02^a^ ± 0.45 log10 CFU/g; experimental: 2.36^b^ ± 0.57 log10 CFU/g) differed significantly between the piglets of both groups in the present study, 28 days after artificial infection [18]. A total of 13 differentially abundant OTUs (*P*-values <0.01; Figure 6) were identified in the pairwise comparisons of pigs having *Salmonella* counts above versus below 3.2 log_10_ CFU/g sample in cecal contents. Of all identified differentially abundant OTUs in cecal contents between both feeding groups, six appeared to be related to *Salmonella* counts as well, under them OTU_23 (genus *Catenisphaera*) and OTU_31 (genus *Bifidobacterium,* Table S8). Both OTUs were enriched in the cecal contents of piglets with *Salmonella* counts below 3.2 log_10_ CFU/g sample compared to piglets with *Salmonella* counts above 3.2 log_10_ CFU/g sample.

## 4. Discussion

### 4.1. Relation between Salmonella Typhimurium and the Differing Bacterial Species

OTU_31 (genus *Bifidobacterium*) and OTU_23 (genus *Catenisphaera*) seemed to be related to *Salmonella* Typhimurium counts in the present study. A high antagonistic activity of *Bifidobacterium* against *Salmonella* was already shown [36,37,38]. To our knowledge, this is the first study to show a relation between the genus *Catenisphaera* and *Salmonella* Typhimurium. The genus *Catenisphaera* was proposed as a member of the family *Erysipelotrichaceae* and has one known species, *Catenisphaera adipataccumulans* [39]. Even if the relative abundance of *Catenisphaera* was on average below 1% in cecal contents as well as in fecal samples of the present study, this genus seems to play a role within the intestinal ecosystem as *Catenisphaera* is found in association with several pig diseases. In F18-fimbriated enterotoxigenic *Escherichia coli* infected weaned pigs, *Catenisphaera* was decreased in the ascending colon [40], while the porcine circovirus type 3 infection caused a higher abundance of *Catenisphaera* [41]. So far, this genus has also been observed in connection with the alleviation of further inflammatory diseases in pigs. Besides increased SCFAs in feces, blood, cecal and colonic digesta, *Catenisphaera* was found to be enriched in the feces of pigs fed a fiber-rich diet (low fiber diet + inulin and cellulose) in association with reduced lung damage/promoted lung recovery [42]. In addition, *Catenisphaera* was identified as the bacterial genera associated with fecal microbiota transplantation that partially relieved the symptoms of postweaning diarrhea in piglets [43]. *Catenisphaera* produce lactate as a major end-product from glucose fermentation [39]. Major fermentation end products of bifidobacteria are acetate and lactate [4,44,45]. Besides the bifidobacterial strain and the strain belonging to the genus *Catenisphaera*, further bacterial strains belonging to the order *Lactobacillales* and genera comprising lactic acid bacteria [34] were enriched in cecal samples of piglets fed the experimental rye-based diet: two of the genus *Lactobacillus* (OTU_115 and OTU_42) and two of the genus *Streptococcus* (OTU_959 and OTU_2). Piglets aged about 47 days that received the same diets as offered in the present study showed significantly higher concentrations of lactic acid in the digesta of the stomach and small intestine, the main sites of lactic acid production [9,46,47], when fed the experimental diet (69% rye) compared to the control diet (69% wheat) [47,48]. In addition, with increasing lactic acid concentrations, the pH tended to be lower in the digesta of the small intestine [47]. Now, we could show in the present study several lactic acid producing bacteria were enriched when feeding the rye-based compared to the wheat-based diet.

The antibacterial activity of several lactobacilli species against *Salmonella* Typhimurium can be solely due to the production of lactic acid, while others inhibit it due to the production of lactic acid and (an) unknown inhibitory substance(s), which are only active in the presence of lactic acid [49,50]. In addition, the produced lactic acid was responsible for a significant inhibitory activity upon invasion of *Salmonella* Typhimurium into cultured human enterocyte-like Caco-2/TC7 cells [49]. However, a translocation of *Salmonella* in ileocecal lymph nodes was not affected by the two diets, as investigated and published by Chuppava, et al. [18] with the pigs of the present study. Even if bifidobacteria produce strong acids, and especially acetate gives a concentration-dependent inhibition of *Salmonella* Typhimurium in vitro [51,52], this SCFA is also known to increase the invasion gene expression and virulence of *Salmonella* Typhimurium [14,53]. SCFA synthesis by the microbiota can be used by the host to induce defense mechanisms but also by the bacterium itself to enhance the invasion of the intestinal epithelium [15]. In addition, a dysbiosis-associated increase in lactate levels also supports *Salmonella* Typhimurium growth, as *Salmonella* Typhimurium utilizes L-lactate as a nutrient during gut infection [54]. Lactate is seldom detected as a major fermentation product of mixed anaerobic communities neither in human feces under normal conditions [55,56], nor in piglet feces although high lactate levels were induced in the small intestine directly or indirectly by feeding measures [47,57]. Piglets that received the same diets as in the present study showed higher concentrations of lactic acid in digesta with decreasing concentrations, the more caudal in the digestive tract the lactate concentrations were examined [47] —an observation which is assumed to reflect absorption and lactate utilization by other bacterial species within a healthy microbiota [55].

Nevertheless, once entering the gastrointestinal tract, *Salmonella enterica* encounters host responses targeted towards pathogen clearance [13]. While the inflammatory response against *Salmonella enterica* has an important role in preventing infection, it also provides the bacteria with unique metabolites such as tetrathionate and is thus exploited by *Salmonella enterica* for its competitive advantage and its ability to outcompete the intestinal microbiota [13,15,58]. In mice, it could be shown that *Salmonella* Typhimurium was unable to colonize the intestine without inflammation [59]. *Salmonella* Typhimurium overcomes colonization resistance by abusing the host’s inflammatory immune response to gain an edge on the normal microbial community of the gut [59,60]. However, immune response is not the main determinant of *Salmonella* serovar Typhimurium levels within the colon, and the intestinal microbiota seem to be involved in the development of the supershedder phenotype [61]. In the natural porcine host as well, microbiota of communities of low and high *Salmonella*-shedding pigs differed [62]. The authors of that study discussed whether differences between the gastrointestinal microbiota of pigs producing a range of SCFA concentrations may influence the acute colonization potential of *Salmonella*, or whether later high shedders have an elevated inflammatory status in the gut that more readily leads to colonization with *Salmonella*, as inflammation is an important component of the pathogenesis strategy of *Salmonella* [62]. Further, studies have shown that the intestinal microbiota of pigs is affected upon exposure to *Salmonella* Typhimurium, and those evaluations revealed an influence on *Faecalibacterium* [63,64]. Besides *Bifidobacterium*, one bacterial species assigned to this genus *Faecalibacterium* was enriched in the cecal contents of piglets fed the experimental rye-based diet. A comparison of the microbiota in the pig colon of challenged pigs and nonchallenged pigs showed a decrease in *Faecalibacterium* at the genus level in *Salmonella* Typhimurium challenged pigs three weeks postchallenge [63]. In addition, the most remarkable difference reported in piglets infected with *Salmonella* Typhimurium compared with the naïve control group was an abundant presence of lactic acid producing bacteria and a reduction of SCFAs-producing bacteria, under them *Ruminococcaceae* including *Faecalibacterium*, *Roseburia*, *Butyrivibrio* and *Clostridium* genera [64]. For this reason, it can be assumed that possible changes in microbiota composition induced by *Salmonella* Typhimurium exposure were mitigated by the experimental diet. However, a negative control group was not considered with the infected groups to be able to prove this effect of the experimental diet with the present study. *Faecalibacterium* has one identified species, *Faecalibacterium prausnitzii* [35], a butyrate-, formate- and D-lactate-producing bacterium [65,66]. *Faecalibacterium prausnitzii* is considered as a beneficial intestinal commensal, and its prevalence is often decreased in conditions of intestinal dysbiosis [65]. Still, little is known about the diversity and functional specificities that are linked to these phylogroups of *Faecalibacterium prausnitzii* [35]. *Faecalibacterium prausnitzii* has been suggested to be a functionally important member of the microbiota and probably has an impact on the physiology and health of the host [35]. *Faecalibacterium prausnitzii* is an acetate-consuming bacterium potentially making a significant contribution to D-lactate and butyrate formation in the large intestine [65,66].

Finally, the microbiota of the large intestine degrade and metabolize dietary fiber not only to SCFAs and gases, but they also convert the phenolic compounds into a range of other metabolites with the ability to influence the metabolism at a cellular level [67]. Not only their metabolites but also the polyphenols themselves can influence and induce a modulation of the gut microbiota composition, thereby affecting the growth of pathogenic and beneficial bacteria and are described as being able to prevent diseases associated with oxidative stress such as inflammatory diseases [68,69].

### 4.2. Differences in Bacterial Composition between Sampling Sites Due to the Feeding Measures

The factor diet had a significant effect on the bacterial composition of the samples at both sampling sites. However, the samples of pigs fed the control or experimental diet separated more clearly in cecal contents (R^2^ = 0.095) with high and continuous supply of fermentable substrates compared to feces (R^2^ = 0.081), where the majority of fermentable substrates had been depleted.

Cereal grains from different species differ significantly in several carbohydrate fractions, while rye has the highest concentrations of soluble arabinoxylans and fructans and is furthermore a valuable source of several bioactive compounds [5,70]. Neither arabinoxylans nor fructans are degraded by endogenous enzymes but with microbial enzymes, thus having a great impact on the site of their digestion/fermentation and how they influence the physiology of the gastrointestinal tract [2]. The majority of soluble arabinoxylans is broken down between the ileum and the cecum [8,46], and arabinoxylans, characterized by a low degree of substitution and virtually no doubly substituted xylose, are slowly degraded at more distal locations, while the remaining arabinoxylans, characterized by a high degree of substitution, are not degraded at all [8]. In addition, a large part of the fructans is already absorbed in the small intestines and the majority disappears within the cecum of pigs [46]. The only bifidobacterial strain found within the porcine microbiota of the present study was significantly enriched only in the cecal samples of pigs fed a diet containing rye instead of wheat. In feces, this difference was no longer seen. The substrate utilization of bifidobacteria from human and animal origin is highly variable and considerable interspecies and interstrain differences exist [71,72,73]. Bifidobacteria are generally able to ferment arabinoxylans; nevertheless, utilization is again limited to only a few bifidobacterial strains [73,74,75]. In addition, cell walls of wheat and rye endosperms are both rich in arabinoxylans, but have structural differences in α-L-arabinofuranosyl substitution patterns and arabinoxylan hydrolysates originating from either rye or wheat as a substrate also induce a strain-dependent intensity and rapidity in growth [73]. Thus, if bifidobacteria had been present in the pigs in our study that were able to use arabinoxylans in the first place, this difference in the structure of arabinoxylans between rye and wheat, may have caused different growth conditions for the bacteria in the different feeding groups. Still, being aware of the limited ability of only a few bifidobacteria to degrade arabinoxylans, it is conceivable that other more versatile polysaccharide-degrading bacteria, such as bacteroides and clostridia, degrade arabinoxylan to smaller molecules, which are then cross-fed to bifidobacteria [8]. Further investigations studying the growth of bifidobacteria, especially on rye- or wheat-originating water-soluble polysaccharides (WSP), speak in favor of the use of other carbohydrates than arabinoxylans. When bifidobacteria were grown on wheat and rye WSP, xylose and arabinose concentrations remained constant, indicating the inability of the tested strains to use arabinoxylan as a carbohydrate source, while polyfructan and a polysaccharide fraction composed of glucose were degraded [44,76]. Fructooligosaccharides or oligofructose are short-chain fructans with a degree of polymerization (DP) lower than 10 [77]. Carbohydrates containing almost exclusively fructose monomers are referred to as fructans [77]. Fructans with β-2,1 glycosidic bonds, called inulin-type fructans, are easily degraded by enzymes, which are frequently found in bacteria of the genus *Bifidobacterium* [77]. Similar to the use of arabinoxylans, there is a specificity of *Bifidobacterium* to utilize short-chain fructooligosaccharides and oligofructose as well as short-chain, but not highly polymerized inulins, while highly polymerized inulins seem to be dependent on the presence and ability of other bacteria to initiate degradation, followed by the possible subsequent stimulation of *Bifidobacterium*‘s growth [78]. Under cereal grains, rye has not only the highest amounts of fructans [5,70], but the fructans of rye and wheat differ in their degree of polymerization. Rye has higher levels of high-molecular-weight fructans (DP > 5) [79], whereas wheat has higher concentrations of fructans of low DP (<5) [80]. Interestingly, bifidobacteria were nevertheless enriched in the cecal contents of pigs when offered the rye-based diet compared to the wheat-based diet. Considering the limited ability of only a few bifidobacteria to degrade arabinoxylans and their specificity to utilize not highly polymerized fructans, the enrichment of *Bifidobacterium* when fed the experimental diet could be explained by the presence of other bacteria within the microbiota that initiate degradation as previously discussed. However, it should also be considered that possibly the quantity of other substrates available for the bacteria within the intestinal tract of pigs differs depending on the diet. The substrates fermented by the largest number of bifidobacterial species were, besides D-galactosamine and D-glucosamine, the two components of starch, amylose and amylopectin [71]. The apparent ileal digestibility (AID) of starch in growing pigs is higher in wheat compared to hybrid rye [10]. However, all grains had high AID values for starch that were above 95% and the total starch contents of wheat were higher compared to that of hybrid rye [10]. Moreover, in the present study, the starch contents of the wheat-based control diet exceeded that of the experimental rye-based diet. Since the AID of starch was not determined in the present study, we cannot give a statement about how much starch entered the cecum and whether these amounts differed between the two different diets. Nevertheless, we already showed in other investigations that *Bifidobacterium* was favored insofar as a higher amount of starch escaped digestion and absorption in the small intestine and reached the large intestine, whereby it became available as a substrate for the present microbiota [57].

As acetate is produced by bifidobacteria when metabolizing carbohydrates originating from rye, this fermentation end-product can additionally be cross-fed. There are several indications of cross-feeding between bifidobacteria and *Faecalibacterium prausnitzii* [65,81,82]. Besides being net acetate utilizers, all *Faecalibacterium prausnitzii* strains possessed the ability to hydrolyze fructose, fructo-oligosaccharide, starch and inulin, but none of the strains utilized arabinose, melibiose, raffinose, rhamnose, ribose and xylose [66]. Thus, the intake of fructan contained in rye may have also directly promoted the growth of this bacterial species. Cross-feeding between bifidobacteria and many other bacteria such as *Faecalibacterium*, which can now use these fermentation end-products and again produce other SCFAs, helps to explain the reported butyrogenic effects of certain dietary substrates [4,55]. In the presence of the bifidobacteria, there is an enhanced formation of butyrate by *Faecalibacterium prausnitzii* [82]. Nielsen et al. [83] observed a higher number of the butyrate-producing *Faecalibacterium prausnitzii* as well as of *Bifidobacterium* spp. and *Lactobacillus* spp. in feces and a three- to fivefold higher pool size of butyrate in the cecum, proximal and mid colon after feeding rye flakes instead of wheat flour to pigs (body weight on average 63 kg) for three weeks. Significantly higher butyrate concentrations were found in the portal vein and mesenteric artery of pigs fed a rye-based instead of a wheat-based diet [9]. Besides those investigations in pigs [83], in human studies as well [84,85], *Faecalibacterium* was already associated with the dietary intake of rye.

In contrast to soluble arabinoxylans and a large part of fructans, NCP-glucose is still largely digested between cecum and feces and its disappearance is still high in the colon, while cellulose seems to disappear only in the colon of pigs fed whole rye [46]. This change in substrate availability between the different sites of the hindgut was reflected by the varying bacterial composition between the cecal contents and feces of the pigs (R^2^ = 0.07573, *p* = 0.001), regardless of the diet that was offered. At this point, it should be emphasized that the bifidogenic effect and growth promotion of several lactobacilli strains as well as of *Faecalibacterium prausnitzii* in pigs fed a rye diet would not have been detected if only the feces had been analyzed. The results obtained in the present study might be of value for extrapolating to humans.

Nevertheless, there were still differences in bacterial composition of feces between the feeding groups of the present study. According to Bach Knudsen et al. [9], the difference in the fermentation pattern (carbohydrate-derived metabolites of bacterial fermentation) in the large intestine of pigs consuming rye compared to wheat are caused by the type rather than the amount of carbohydrate entering the large intestine. Comparable to the investigations of Bach Knudsen et al. [9], the most noticeable difference between the two diets was the nature of the NSP fractions. The wheat-based diet had a high proportion of NSP as cellulose, whereas the rye-based diet had a high content of total and soluble arabinoxylans as well as β-glucan. The digestibility of β-glucan before reaching the ileum of pigs offered a wheat-based diet was higher compared to a rye-based diet, whereas the disappearance of β-glucan between cecum and rectum was lower for the wheat-based than the rye-based diet, 34% for the wheat-based compared to 51% for the rye-based diet [9]. Almost none of the β-glucan offered with the rye-based diet was excreted via feces (fecal digestibility of 99.2%) [9]. In the case of cellulose, ileal digestibility did not differ between the rye-based and wheat-based diets (wheat: 25.7% ± 1.5 vs. rye: 26.8% ± 1.3). However, fecal digestibility of cellulose was numerically higher when wheat was offered (wheat: 36.3% ± 4.0 vs. rye: 28.5% ± 4.0) [9].

## 5. Conclusions

Dietary measures contributed significantly to the differences of microbial composition, while samples separated more clearly in cecal contents compared to feces by this variable alone. In the cecum, a promoted growth of *Bifidobacterium*, several lactic acid bacteria and *Faecalibacterium prausnitzii* were seen in pigs fed the experimental diet. The high intake of rye instead of wheat seems to promote the growth of beneficial intestinal bacteria accompanied by impaired growth conditions for the foodborne pathogen *Salmonella* Typhimurium.

## Figures and Tables

**Table 1:** Ingredients and chemical composition of compound feeds (g/kg dry matter basis).

Ingredients	Control Diet (69% Wheat)	Experimental Diet (69% Rye)
Wheat	69.0%	-
Rye	-	69.0%
Soybean meal	11.5%	11.5%
Barley	10.0%	10.0%
Potato protein	5.10%	4.90%
Calcium carbonate	1.00%	0.90%
Monocalcium phosphate	0.90%	1.00%
Soybean oil	0.50%	0.50%
Sodium chloride	0.35%	0.40%
Feed additives *	1.65%	1.80%
**Chemical composition**		
Dry matter (g/kg as fed)	897	903
Crude ash	51.4	54.2
Crude protein	205	213
Ether extract	28.0	23.6
Crude fiber	28.7	28.2
Starch	530	491
Metabolizable energy (ME, MJ/kg DM)	15.6	15.5

* Feed additives (per kg as fed): vitamin A (12,000 IU), vitamin D3 (2000 IU), vitamin E (150 mg), copper from copper-(II)-glycinate chelate hydrate (4 mg) and copper-(II)-sulfate pentahydrate (110 mg), manganese from manganese glycine manganese chelate hydrate (35 mg), manganese from manganese-(II)-oxide (45 mg), zinc from glycine zinc chelate hydrate (40 mg) and zinc oxide (80 mg), iron from iron-(II)-sulfate monohydrate (200 mg), iodine from calcium iodate anhydrous (2.0 mg) and selenium from sodium selenite (0.40 mg); zootechnical additives: 5.0 × 10^9^ CFU *Saccharomyces cerevisiae*.

**Table 2:** Carbohydrate composition of compound feeds (%, dry matter basis) ^1^.

	Control Diet (69% Wheat)	Experimental Diet (69% Rye)
Dry matter (% as fed)	92.1	92.3
S-NCP ^2^	3.5	4.6
Arabinose	0.8	1.2
Xylose	1.0	1.5
Mannose	0.1	0.2
Galactose	0.5	0.4
Glucose	0.8	1.0
Uronic acids	0.3	0.3
I-NCP ^3^	6.5	7.5
Arabinose	1.7	1.8
Xylose	2.9	2.9
Mannose	0.3	0.4
Galactose	0.4	0.4
Glucose	0.7	1.6
Uronic acids	0.5	0.4
Cellulose	2.4	1.8
NSP (total) ^4^	12.3	14.0
Klason lignin	2.0	1.6
Soluble dietary fiber ^5^	3.5	4.6
Insoluble dietary fiber ^6^	10.8	10.9
Dietary fiber ^7^	14.3	15.6

^1^ Rhamnose and fucose in both compound feeds were not detected and therefore excluded from the table. ^2^ S-NCP = soluble noncellulosic polysaccharides. ^3^ I-NCP = insoluble noncellulosic polysaccharides. ^4^ NSP = nonstarch polysaccharides. Total NSP = S-NCP + I-NCP + cellulose. Smaller deviations in the sum after the decimal point are due to rounding. ^5^ Soluble dietary fiber = S-NCP. ^6^ Insoluble dietary fiber = I-NCP + cellulose + Klason lignin. Smaller deviations in the sum after the decimal point are due to rounding. ^7^ Dietary fiber = soluble dietary fiber + insoluble dietary fiber.

## Data Availability

The datasets for this study can be found in the NCBI BioProject with the accession ID PRJNA841815.

## Supplementary Materials

The following supporting information can be downloaded at: https://www.mdpi.com/article/10.3390/plants11172232/s1, Figure S1: Bray–Curtis dissimilarity-based principal coordinate analysis (PCoA) was performed on samples of pigs fed with either rye- or wheat-containing diets. Different colors represent samples obtained from different parts (cecal contents or feces). Lines connect samples obtained from the same site; Table S1: Alpha diversity (means ± SD) in samples using the species richness estimators Observed species, Chao1, and Shannon index; Table S2: Permutational multivariate analysis of variance (PERMANOVA) results based on Bray–Curtis dissimilarities, Table S3: Differentially abundant OTUs in the pairwise comparisons of the groups in cecal contents, Table S4: Differentially abundant OTUs in the pairwise comparisons of the groups in fecal samples, Table S5: Differentially abundant OTUs in the pairwise comparisons of the groups in cecal contents of experiment 1, Table S6: Differentially abundant OTUs in the pairwise comparisons of the groups in cecal contents of experiment 2, Table S7: Differentially abundant OTUs in the pairwise comparisons of the groups in cecal contents of experiment 3, Table S8: Differentially abundant OTUs in the pairwise comparison of piglets with *Salmonella* counts above versus below 3.2 log_10_ CFU/g cecal content.
