# Supplementary material for: High Dietary Intake of Rye Affects Porcine Gut Microbiota in a Salmonella Typhimurium Infection Study
Source: Plants (Basel). 2022 Aug 28;11(17):2232. doi: 10.3390/plants11172232 (PMC9460007; doi:10.3390/plants11172232)
Supplement: Supplementary file 1 [file plants-11-02232-s001.zip › plants-1877622-supplementary.pdf]

Supplementary Material

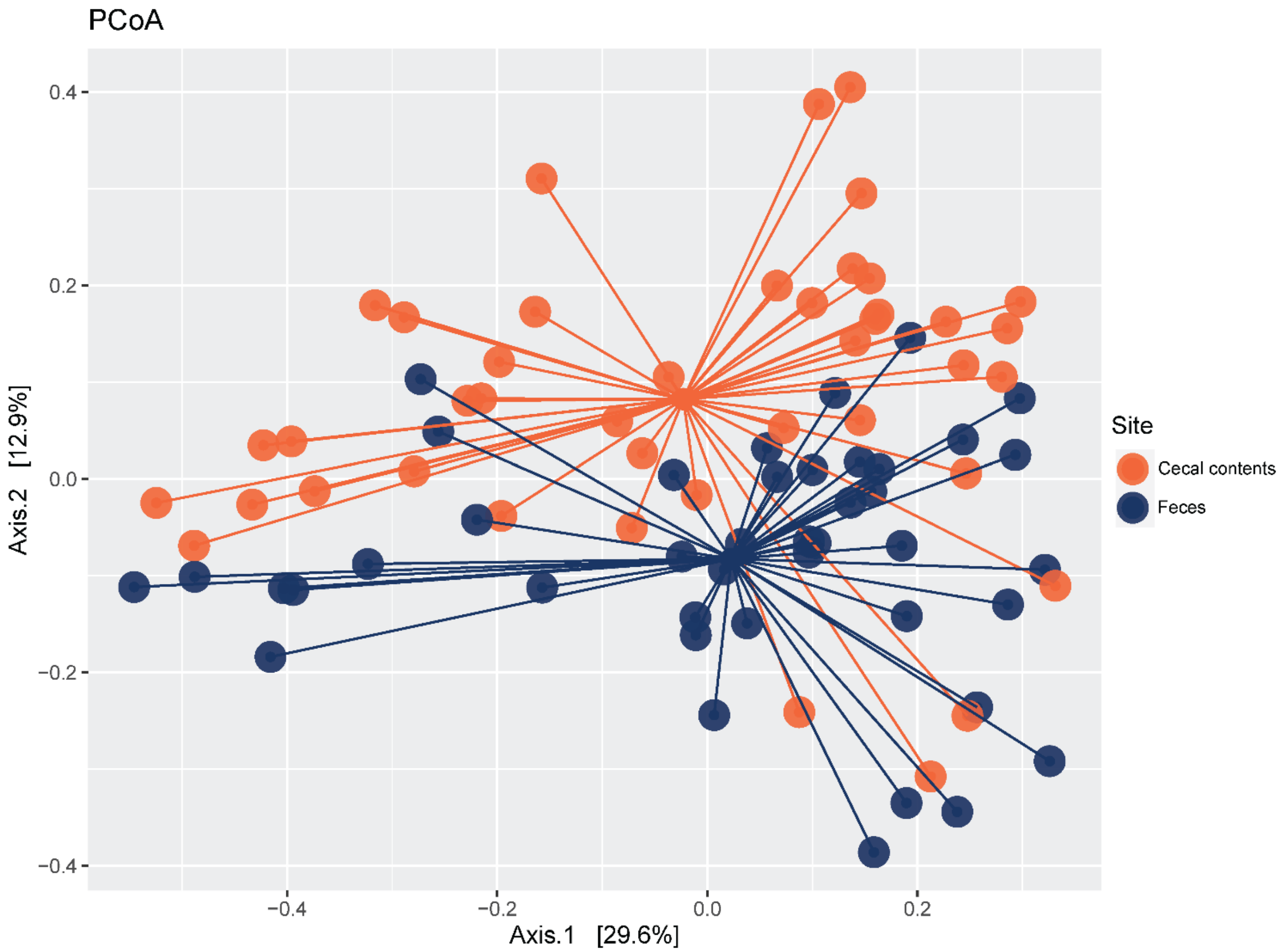

Figure S1. Bray-Curtis dissimilarity-based principal coordinate analysis (PCoA) was performed on samples of pigs fed with either rye- or wheat-containing diets. Different colors represent samples obtained from different parts (cecal contents or feces). Lines connect samples obtained from the same site.

Table S1. Alpha diversity (means ± SD) in samples using the species richness estimators Observed species, Chao1, and Shannon index.

	**Cecal contents**		***P*-value**	**Feces**		***P*-value**
	**Rye, n=21**	**Wheat, n=21**		**Rye, n=21**	**Wheat, n=21**	
Observed	155 ±31.2	163 ±26.9	0.326	192 ±21.4	193 ±27.8	0.536
Chao1	176 ±36.3	183 ±30.4	0.508	207 ±23.0	207 ±28.9	0.811
Shannon	3.02 ±0.332	3.13 ±0.313	0.253	3.33 ±0.255	3.50 ±0.379	0.089

Table S2. Permutational multivariate analysis of variance (PERMANOVA) results based on Bray- Curtis dissimilarities.

**Cecal contents**	**DF**	**SumsOfSqs**	**F.Model**	**R2**	**Pr(>F)**
Experiment	2	1.0444	3.4338	0.13851	0.002 **
Diet	1	0.7172	4.7156	0.09511	0.002 **
Residuals	38	5.7792		0.76639	
Total	41	7.5408		1.00000	
**Feces**					
Experiment	2	1.1062	3.5634	0.14517	0.002 **
Diet	1	0.6157	3.9668	0.08080	0.002 **
Residuals	38	5.8981		0.77403	
Total	41	7.6200		1.00000	

**: *P*-value < 0.01.

Table S3. Differentially abundant OTUs in the pairwise comparisons of the groups (rye vs wheat) in cecal contents.

	log2FoldChange	pvalue	padj	Phylum	Class	Order	Family	Genus
OTU_31	4.39	1.52E-06	0.0003	Actinobacteria	Actinobacteria	Bifidobacteriales	Bifidobacteriaceae	Bifidobacterium
OTU_101	-3.28	3.35E-06	0.0003	Firmicutes	Clostridia	Clostridiales	Peptostreptococcaceae	Romboutsia
OTU_16	-2.30	9.56E-06	0.0006	Firmicutes	Erysipelotrichia	Erysipelotrichales	Erysipelotrichaceae	Turicibacter
OTU_6	-1.62	0.0001	0.0022	Firmicutes	Clostridia	Clostridiales	Peptostreptococcaceae	Terrisporobacter
OTU_23	2.87	0.0001	0.0022	Firmicutes	Erysipelotrichia	Erysipelotrichales	Erysipelotrichaceae	Catenisphaera
OTU_115	3.22	0.0001	0.0022	Firmicutes	Bacilli	Lactobacillales	Lactobacillaceae	Lactobacillus
OTU_82	-5.64	0.0002	0.0049	Bacteroidetes	Bacteroidia	Bacteroidales	Prevotellaceae	Ambiguous_taxa
OTU_43	2.32	0.0003	0.0073	Firmicutes	Bacilli	Lactobacillales	Lactobacillaceae	Lactobacillus
OTU_959	2.32	0.0004	0.0073	Firmicutes	Bacilli	Lactobacillales	Streptococcaceae	Streptococcus
OTU_189	-2.75	0.0006	0.0103	Firmicutes	Clostridia	Clostridiales	Christensenellaceae	Christensenellaceae R-7 group
OTU_2	1.66	0.0013	0.0220	Firmicutes	Bacilli	Lactobacillales	Streptococcaceae	Streptococcus
OTU_26	1.67	0.0016	0.0254	Firmicutes	Erysipelotrichia	Erysipelotrichales	Erysipelotrichaceae	Catenibacterium
OTU_575	-2.27	0.0021	0.0296	Firmicutes	Clostridia	Clostridiales	Clostridiaceae 1	Clostridium sensu stricto 1
OTU_40	1.02	0.0025	0.0304	Firmicutes	Clostridia	Clostridiales	Ruminococcaceae	Faecalibacterium
OTU_171	-2.82	0.0026	0.0304	Firmicutes	Clostridia	Clostridiales	Lachnospiraceae	[Eubacterium] hallii group
OTU_47	-4.10	0.0026	0.0304	Firmicutes	Clostridia	Clostridiales	Lachnospiraceae	Blautia
OTU_428	-1.65	0.0028	0.0304	Firmicutes	Clostridia	Clostridiales	Lachnospiraceae	[Ruminococcus] torques group
OTU_638	-3.35	0.0032	0.0320	Firmicutes	Clostridia	Clostridiales	Peptococcaceae	Peptococcus
OTU_13	1.80	0.0033	0.0320	Bacteroidetes	Bacteroidia	Bacteroidales	Prevotellaceae	Ambiguous_taxa
OTU_8	1.53	0.0039	0.0361	Firmicutes	Negativicutes	Selenomonadales	Veillonellaceae	Dialister
OTU_75	5.85	0.0055	0.0485	Firmicutes	Erysipelotrichia	Erysipelotrichales	Erysipelotrichaceae	Solobacterium

Raw *P*-values were adjusted using the method of Benjamini and Hochberg [32] to control a false discovery rate (FDR) of 5%.

Table S4. Differentially abundant OTUs in the pairwise comparisons of the groups (rye vs wheat) in fecal samples.

	log2FoldChange	pvalue	padj	Phylum	Class	Order	Family	Genus
OTU_23	3.29	1.02E-07	3.19E-05	Firmicutes	Erysipelotrichia	Erysipelotrichales	Erysipelotrichaceae	Catenisphaera
OTU_101	-3.32	0.0001	0.0165	Firmicutes	Clostridia	Clostridiales	Peptostreptococcaceae	Romboutsia
OTU_6	-1.83	0.0002	0.0228	Firmicutes	Clostridia	Clostridiales	Peptostreptococcaceae	Terrisporobacter
OTU_16	-2.08	0.0004	0.0285	Firmicutes	Erysipelotrichia	Erysipelotrichales	Erysipelotrichaceae	Turicibacter
OTU_66	-1.21	0.0006	0.0305	Firmicutes	Clostridia	Clostridiales	Family XIII	Mogibacterium
OTU_821	2.08	0.0008	0.0305	Bacteroidetes	Bacteroidia	Bacteroidales	Prevotellaceae	Prevotella 9
OTU_19	-1.57	0.0008	0.0305	Firmicutes	Clostridia	Clostridiales	Ruminococcaceae	Subdoligranulum
OTU_64	-1.28	0.0008	0.0305	Bacteroidetes	Bacteroidia	Bacteroidales	Rikenellaceae	Rikenellaceae RC9 gut group
OTU_362	-1.11	0.0009	0.0305	Firmicutes	Clostridia	Clostridiales	Lachnospiraceae	Ambiguous_taxa
OTU_68	3.75	0.0013	0.0360	Firmicutes	Clostridia	Halanaerobiales	ODP1230B8.23	Ambiguous_taxa
OTU_13	1.81	0.0013	0.0360	Bacteroidetes	Bacteroidia	Bacteroidales	Prevotellaceae	Ambiguous_taxa
OTU_50	-0.82	0.0014	0.0360	Firmicutes	Clostridia	Clostridiales	Lachnospiraceae	[Ruminococcus] gauvreauii group
OTU_2	1.43	0.0016	0.0360	Firmicutes	Bacilli	Lactobacillales	Streptococcaceae	Streptococcus
OTU_63	-1.72	0.0016	0.0360	Firmicutes	Erysipelotrichia	Erysipelotrichales	Erysipelotrichaceae	Solobacterium
OTU_90	-1.42	0.0024	0.0491	Firmicutes	Erysipelotrichia	Erysipelotrichales	Erysipelotrichaceae	Holdemanella

Raw *P*-values were adjusted using the method of Benjamini and Hochberg [32] to control a false discovery rate (FDR) of 5%.

Table S5. Differentially abundant OTUs in the pairwise comparisons of the groups (rye vs wheat) in cecal contents of experiment 1.

	log2FoldChange	pvalue	padj	Phylum	Class	Order	Family	Genus
OTU_1	-4.80	4.8E-05	0.0126	Firmicutes	Clostridia	Clostridiales	Clostridiaceae 1	Clostridium sensu stricto 1
OTU_6	-2.85	0.0003	0.0443	Firmicutes	Clostridia	Clostridiales	Peptostreptococcaceae	Terrisporobacter
OTU_428	-3.34	0.0013	0.1133	Firmicutes	Clostridia	Clostridiales	Lachnospiraceae	[Ruminococcus] torques group
OTU_959	2.84	0.0018	0.1133	Firmicutes	Bacilli	Lactobacillales	Streptococcaceae	Streptococcus
OTU_94	5.98	0.0022	0.1133	Bacteroidetes	Bacteroidia	Bacteroidales	Prevotellaceae	Prevotella 7
OTU_197	5.22	0.0048	0.2083	Bacteroidetes	Bacteroidia	Bacteroidales	Bacteroidales S24-7 group	Ambiguous_taxa
OTU_211	-6.39	0.0075	0.2189	Bacteroidetes	Bacteroidia	Bacteroidales	Bacteroidales S24-7 group	Ambiguous_taxa
OTU_39	-3.22	0.0077	0.2189	Firmicutes	Clostridia	Clostridiales	Lachnospiraceae	[Ruminococcus] gauvreauii group
OTU_189	-3.90	0.0078	0.2189	Firmicutes	Clostridia	Clostridiales	Christensenellaceae	Christensenellaceae R-7 group
OTU_57	-4.25	0.0084	0.2189	Firmicutes	Clostridia	Clostridiales	Ruminococcaceae	Ruminococcus 2
OTU_243	-3.42	0.0119	0.2824	Firmicutes	Clostridia	Clostridiales	Lachnospiraceae	Roseburia
OTU_115	3.04	0.0133	0.2897	Firmicutes	Bacilli	Lactobacillales	Lactobacillaceae	Lactobacillus
OTU_37	-2.38	0.0204	0.3861	Firmicutes	Bacilli	Lactobacillales	Lactobacillaceae	Lactobacillus
OTU_34	-2.23	0.0225	0.3861	Firmicutes	Clostridia	Clostridiales	Ruminococcaceae	Ruminococcaceae UCG-005
OTU_75	4.61	0.0243	0.3861	Firmicutes	Erysipelotrichia	Erysipelotrichales	Erysipelotrichaceae	Solobacterium
OTU_129	-5.25	0.0246	0.3861	Firmicutes	Erysipelotrichia	Erysipelotrichales	Erysipelotrichaceae	Catenisphaera
OTU_184	-4.01	0.0251	0.3861	Bacteroidetes	Bacteroidia	Bacteroidales	Prevotellaceae	Alloprevotella
OTU_27	1.73	0.0329	0.4567	Firmicutes	Clostridia	Clostridiales	Lachnospiraceae	Ambiguous_taxa
OTU_105	-2.93	0.0331	0.4567	Proteobacteria	Gammaproteo-bacteria	Pasteurellales	Pasteurellaceae	Actinobacillus
OTU_85	5.46	0.0380	0.4831	Firmicutes	Clostridia	Clostridiales	Lachnospiraceae	Syntrophococcus
OTU_123	-3.12	0.0391	0.4831	Firmicutes	Clostridia	Clostridiales	Lachnospiraceae	[Eubacterium] hallii group
OTU_147	3.46	0.0407	0.4831	Cyanobacteria	Melainabacteria	Gastranaerophilales	Ambiguous_taxa	Ambiguous_taxa
OTU_23	1.84	0.0468	0.4831	Firmicutes	Erysipelotrichia	Erysipelotrichales	Erysipelotrichaceae	Catenisphaera
OTU_104	-5.44	0.0474	0.4831	Firmicutes	Clostridia	Clostridiales	Ruminococcaceae	Ambiguous_taxa
OTU_493	-2.28	0.0476	0.4831	Firmicutes	Clostridia	Clostridiales	Lachnospiraceae	Ambiguous_taxa
OTU_79	-5.46	0.0479	0.4831	Bacteroidetes	Bacteroidia	Bacteroidales	Prevotellaceae	Alloprevotella

Raw *P*-values were adjusted using the method of Benjamini and Hochberg [32] to control a false discovery rate (FDR) of 5%.

Table S6. Differentially abundant OTUs in the pairwise comparisons of the groups (rye vs wheat) in cecal contents of experiment 2.

	log2FoldChange	pvalue	padj	Phylum	Class	Order	Family	Genus
OTU_177	-23.2	8.0E-15	1.8E-12	Bacteroidetes	Bacteroidia	Bacteroidales	Prevotellaceae	Alloprevotella
OTU_52	-22.1	1.4E-13	1.6E-11	Bacteroidetes	Bacteroidia	Bacteroidales	Bacteroidales S24-7 group	Ambiguous_taxa
OTU_31	5.28	1.6E-05	0.0012	Actinobacteria	Actinobacteria	Bifidobacteriales	Bifidobacteriaceae	Bifidobacterium
OTU_101	-3.47	0.0001	0.0061	Firmicutes	Clostridia	Clostridiales	Peptostreptococcaceae	Romboutsia
OTU_47	-8.69	0.0001	0.0065	Firmicutes	Clostridia	Clostridiales	Lachnospiraceae	Blautia
OTU_82	-11.0	0.0002	0.0069	Bacteroidetes	Bacteroidia	Bacteroidales	Prevotellaceae	Ambiguous_taxa
OTU_90	1.65	0.0003	0.0094	Firmicutes	Erysipelotrichia	Erysipelotrichales	Erysipelotrichaceae	Holdemanella
OTU_638	-6.66	0.0007	0.0182	Firmicutes	Clostridia	Clostridiales	Peptococcaceae	Peptococcus
OTU_183	-4.55	0.0007	0.0183	Bacteroidetes	Bacteroidia	Bacteroidales	Porphyromonadaceae	Parabacteroides
OTU_152	-9.03	0.0013	0.0294	Bacteroidetes	Bacteroidia	Bacteroidales	Prevotellaceae	Prevotellaceae UCG-001
OTU_23	3.35	0.0023	0.0460	Firmicutes	Erysipelotrichia	Erysipelotrichales	Erysipelotrichaceae	Catenisphaera
OTU_192	-5.53	0.0026	0.0491	Firmicutes	Clostridia	Clostridiales	Ruminococcaceae	Ambiguous_taxa
OTU_170	2.30	0.0041	0.0696	Firmicutes	Clostridia	Clostridiales	Ruminococcaceae	Ruminiclostridium 5
OTU_182	-3.87	0.0044	0.0701	Firmicutes	Clostridia	Clostridiales	Lachnospiraceae	[Eubacterium] xylanophilum group
OTU_58	1.83	0.0056	0.0801	Firmicutes	Clostridia	Clostridiales	Lachnospiraceae	Dorea
OTU_580	-8.25	0.0057	0.0801	Firmicutes	Clostridia	Clostridiales	Ruminococcaceae	Ruminococcaceae UCG-005
OTU_885	-4.78	0.0072	0.0927	Firmicutes	Clostridia	Clostridiales	Lachnospiraceae	[Eubacterium] oxidoreducens group
OTU_764	-7.26	0.0075	0.0927	Firmicutes	Clostridia	Clostridiales	Lachnospiraceae	[Eubacterium] xylanophilum group
OTU_124	4.66	0.0087	0.1018	Firmicutes	Clostridia	Clostridiales	Ruminococcaceae	[Eubacterium] coprostanoligenes group
OTU_262	-7.47	0.0124	0.1251	Firmicutes	Clostridia	Clostridiales	Ruminococcaceae	[Eubacterium] coprostanoligenes group
OTU_188	-7.42	0.0130	0.1251	Spirochaetae	Spirochaetes	Spirochaetales	Spirochaetaceae	Treponema 2
OTU_272	-7.40	0.0133	0.1251	Firmicutes	Clostridia	Clostridiales	Lachnospiraceae	Acetitomaculum
OTU_44	-4.45	0.0135	0.1251	Bacteroidetes	Bacteroidia	Bacteroidales	Prevotellaceae	Prevotella 1
OTU_687	-7.35	0.0139	0.1251	Bacteroidetes	Bacteroidia	Bacteroidales	Prevotellaceae	Prevotellaceae NK3B31 group
OTU_239	-6.77	0.0145	0.1251	Bacteroidetes	Bacteroidia	Bacteroidales	Bacteroidales S24-7 group	Ambiguous_taxa

	log2FoldChange	pvalue	padj	Phylum	Class	Order	Family	Genus
OTU_141	-6.63	0.0149	0.1251	Tenericutes	Mollicutes	Mollicutes RF9	Ambiguous_taxa Firmicutes bacterium	Ambiguous_taxa Firmicutes bacterium
OTU_301	-6.46	0.0151	0.1251	Firmicutes	Clostridia	Clostridiales	Lachnospiraceae	Lachnospiraceae NK4A136 group
OTU_118	-6.57	0.0158	0.1256	Proteobacteria	Betaproteobacteria	Neisseriales	Neisseriaceae	Leeia
OTU_1267	-3.27	0.0170	0.1287	Bacteroidetes	Bacteroidia	Bacteroidales	Prevotellaceae	Ambiguous_taxa
OTU_198	-6.16	0.0173	0.1287	Bacteroidetes	Bacteroidia	Bacteroidales	Rikenellaceae	Rikenellaceae RC9 gut group
OTU_109	-5.54	0.0180	0.1295	Bacteroidetes	Bacteroidia	Bacteroidales	Prevotellaceae	Prevotellaceae NK3B31 group
OTU_66	-1.45	0.0189	0.1314	Firmicutes	Clostridia	Clostridiales	Family XIII	Mogibacterium
OTU_810	-4.15	0.0223	0.1508	Firmicutes	Clostridia	Clostridiales	Ambiguous_taxa	Ambiguous_taxa
OTU_40	1.18	0.0248	0.1599	Firmicutes	Clostridia	Clostridiales	Ruminococcaceae	Faecalibacterium
OTU_171	-3.41	0.0251	0.1599	Firmicutes	Clostridia	Clostridiales	Lachnospiraceae	[Eubacterium] hallii group
OTU_2	1.83	0.0290	0.1750	Firmicutes	Bacilli	Lactobacillales	Streptococcaceae	Streptococcus
OTU_26	1.70	0.0290	0.1750	Firmicutes	Erysipelotrichia	Erysipelotrichales	Erysipelotrichaceae	Catenibacterium
OTU_72	1.09	0.0305	0.1788	Firmicutes	Clostridia	Clostridiales	Lachnospiraceae	Lachnospiraceae ND3007 group
OTU_110	1.13	0.0334	0.1875	Firmicutes	Clostridia	Clostridiales	Ruminococcaceae	Butyricicoccus
OTU_127	-2.08	0.0336	0.1875	Bacteroidetes	Bacteroidia	Bacteroidales	Prevotellaceae	Ambiguous_taxa
OTU_35	-1.63	0.0377	0.2048	Firmicutes	Negativicutes	Selenomonadales	Acidaminococcaceae	Acidaminococcus
OTU_214	-6.13	0.0404	0.2147	Firmicutes	Clostridia	Clostridiales	Lachnospiraceae	Ambiguous_taxa

Raw *P*-values were adjusted using the method of Benjamini and Hochberg [32] to control a false discovery rate (FDR) of 5%.

Table S7. Differentially abundant OTUs in the pairwise comparisons of the groups (rye vs wheat) in cecal contents of experiment 3.

	log2FoldChange	pvalue	padj	Phylum	Class	Order	Family	Genus
OTU_94	23.6	2.5E-15	7.4E-13	Bacteroidetes	Bacteroidia	Bacteroidales	Prevotellaceae	Prevotella 7
OTU_101	-7.49	1.0E-07	1.6E-05	Firmicutes	Clostridia	Clostridiales	Peptostreptococcaceae	Romboutsia
OTU_31	8.68	1.5E-05	0.0014	Actinobacteria	Actinobacteria	Bifidobacteriales	Bifidobacteriaceae	Bifidobacterium
OTU_115	6.12	4.8E-05	0.0035	Firmicutes	Bacilli	Lactobacillales	Lactobacillaceae	Lactobacillus
OTU_16	-4.07	0.0001	0.0085	Firmicutes	Erysipelotrichia	Erysipelotrichales	Erysipelotrichaceae	Turicibacter
OTU_43	3.36	0.0003	0.0149	Firmicutes	Bacilli	Lactobacillales	Lactobacillaceae	Lactobacillus
OTU_37	4.26	0.0006	0.0267	Firmicutes	Bacilli	Lactobacillales	Lactobacillaceae	Lactobacillus
OTU_575	-4.62	0.0015	0.0567	Firmicutes	Clostridia	Clostridiales	Clostridiaceae 1	Clostridium sensu stricto 1
OTU_23	3.69	0.0025	0.0837	Firmicutes	Erysipelotrichia	Erysipelotrichales	Erysipelotrichaceae	Catenisphaera
OTU_13	2.98	0.0048	0.1422	Bacteroidetes	Bacteroidia	Bacteroidales	Prevotellaceae	Ambiguous_taxa
OTU_177	-6.40	0.0061	0.1590	Bacteroidetes	Bacteroidia	Bacteroidales	Prevotellaceae	Alloprevotella
OTU_964	-3.13	0.0065	0.1590	Firmicutes	Negativicutes	Selenomonadales	Veillonellaceae	Anaerovibrio
OTU_6	-2.09	0.0074	0.1590	Firmicutes	Clostridia	Clostridiales	Peptostreptococcaceae	Terrisporobacter
OTU_159	-4.64	0.0075	0.1590	Firmicutes	Clostridia	Clostridiales	Lachnospiraceae	[Eubacterium] ruminantium group
OTU_664	-5.67	0.0082	0.1625	Bacteroidetes	Bacteroidia	Bacteroidales	Porphyromonadaceae	Parabacteroides
OTU_54	-4.11	0.0088	0.1634	Bacteroidetes	Bacteroidia	Bacteroidales	Prevotellaceae	Prevotellaceae NK3B31 group
OTU_83	1.38	0.0105	0.1823	Firmicutes	Clostridia	Clostridiales	Lachnospiraceae	Dorea
OTU_165	-7.19	0.0153	0.2517	Spirochaetae	Spirochaetes	Spirochaetales	Spirochaetaceae	Treponema 2
OTU_638	-2.13	0.0162	0.2524	Firmicutes	Clostridia	Clostridiales	Peptococcaceae	Peptococcus
OTU_48	3.04	0.0188	0.2776	Bacteroidetes	Bacteroidia	Bacteroidales	Prevotellaceae	Prevotella 7
OTU_2	1.97	0.0263	0.3711	Firmicutes	Bacilli	Lactobacillales	Streptococcaceae	Streptococcus
OTU_76	-1.57	0.0301	0.4055	Firmicutes	Clostridia	Clostridiales	Peptostreptococcaceae	Ambiguous_taxa
OTU_428	-2.31	0.0389	0.4878	Firmicutes	Clostridia	Clostridiales	Lachnospiraceae	[Ruminococcus] torques group
OTU_269	-2.57	0.0400	0.4878	Firmicutes	Clostridia	Clostridiales	Lachnospiraceae	Lachnospiraceae AC2044 group
OTU_34	-2.10	0.0458	0.4878	Firmicutes	Clostridia	Clostridiales	Ruminococcaceae	Ruminococcaceae UCG-005
OTU_821	4.66	0.0459	0.4878	Bacteroidetes	Bacteroidia	Bacteroidales	Prevotellaceae	Prevotella 9
OTU_296	-3.64	0.0469	0.4878	Firmicutes	Clostridia	Clostridiales	Lachnospiraceae	Butyrivibrio
OTU_49	0.80	0.0476	0.4878	Firmicutes	Clostridia	Clostridiales	Lachnospiraceae	Blautia
OTU_66	-1.16	0.0478	0.4878	Firmicutes	Clostridia	Clostridiales	Family XIII	Mogibacterium

Raw *P*-values were adjusted using the method of Benjamini and Hochberg [32] to control a false discovery rate (FDR) of 5%.

Table S8. Differentially abundant OTUs in the pairwise comparisons of piglets with *Salmonella* counts above versus below 3.2 log_{10} CFU/g cecal content.

	**log2FoldChange**	**pvalue**	**padj**	**Phylum**	**Class**	**Order**	**Family**	**Genus**
OTU_82	5.96	7.17E-05	0.0125	Bacteroidetes	Bacteroidia	Bacteroidales	Prevotellaceae	Ambiguous_taxa
OTU_23	-2.85	7.98E-05	0.0125	Firmicutes	Erysipelotrichia	Erysipelotrichales	Erysipelotrichaceae	Catenisphaera
OTU_638	4.07	0.0002	0.0216	Firmicutes	Clostridia	Clostridiales	Peptococcaceae	Peptococcus
OTU_31	-3.32	0.0008	0.0629	Actinobacteria	Actinobacteria	Bifidobacteriales	Bifidobacteriaceae	Bifidobacterium
OTU_94	-5.82	0.0014	0.0843	Bacteroidetes	Bacteroidia	Bacteroidales	Prevotellaceae	Prevotella 7
OTU_21	-6.37	0.0016	0.0843	Firmicutes	Clostridia	Clostridiales	Lachnospiraceae	Lachnospiraceae NK3A20 group
OTU_26	-1.55	0.0038	0.1715	Firmicutes	Erysipelotrichia	Erysipelotrichales	Erysipelotrichaceae	Catenibacterium
OTU_58	-1.09	0.0055	0.2004	Firmicutes	Clostridia	Clostridiales	Lachnospiraceae	Dorea
OTU_75	-5.80	0.0057	0.2004	Firmicutes	Erysipelotrichia	Erysipelotrichales	Erysipelotrichaceae	Solobacterium
OTU_67	-5.55	0.0074	0.2135	Actinobacteria	Coriobacteriia	Coriobacteriales	Coriobacteriaceae	Olsenella
OTU_53	-0.91	0.0075	0.2135	Firmicutes	Clostridia	Clostridiales	Peptococcaceae	Peptococcus
OTU_109	3.91	0.0084	0.2196	Bacteroidetes	Bacteroidia	Bacteroidales	Prevotellaceae	Prevotellaceae NK3B31 group
OTU_63	-1.33	0.0099	0.2297	Firmicutes	Erysipelotrichia	Erysipelotrichales	Erysipelotrichaceae	Solobacterium

Raw *P*-values were adjusted using the method of Benjamini and Hochberg [32] to control a false discovery rate (FDR) of 5%.
